# Supplementary material for: Inflammatory factors and risk of lung adenocarcinoma: a Mendelian randomization study mediated by blood metabolites
Source: Front Endocrinol (Lausanne). 2024 Aug 27;15:1446863. doi: 10.3389/fendo.2024.1446863 (PMC11384989; doi:10.3389/fendo.2024.1446863)
Supplement: Supplementary file 1 [file DataSheet1.zip › supplementary files/S2/GCST90200853/forest.pdf]

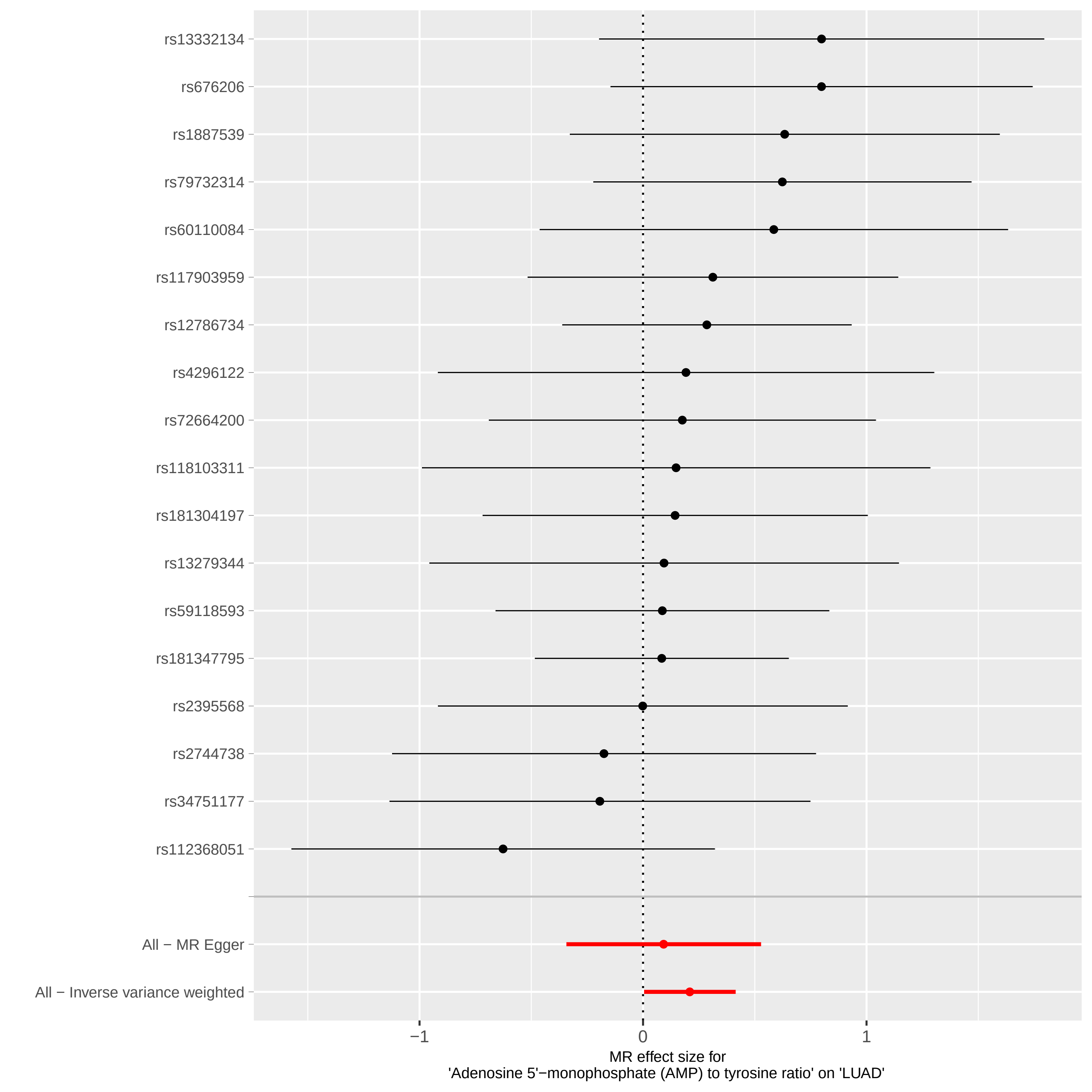
rs13332134
rs676206
rs1887539
rs79732314
rs60110084
rs117903959
rs12786734
rs4296122
rs72664200
rs118103311
rs181304197
rs13279344
rs59118593
rs181347795
rs2395568
rs2744738
rs34751177
rs112368051
All − MR Egger
All − Inverse variance weighted
−1
0
1
MR effect size for
'Adenosine 5'−monophosphate (AMP) to tyrosine ratio' on 'LUAD'
